# Nanocarrier-mediated RNAi of *CYP9A306* and *CYB5R* enhances susceptibility of invasive tomato pest, *Tuta absoluta* to cyantraniliprole

**DOI:** 10.3389/fpls.2025.1573634

**Published:** 2025-04-28

**Authors:** Farman Ullah, Guru-Pirasanna-Pandi G, Hina Gul, Rudra Madhab Panda, Ghulam Murtaza, Zhijun Zhang, Jun Huang, Xiaowei Li, Nicolas Desneux, Yaobin Lu

**Affiliations:** ^1^ State Key Laboratory for Quality and Safety of Agro-Products, Key Laboratory of Biotechnology in Plant Protection of MOA of China and Zhejiang Province, Institute of Plant Protection and Microbiology, Zhejiang Academy of Agricultural Sciences, Hangzhou, China; ^2^ Protection Division, ICAR-National Rice Research Institute, Cuttack, Odisha, India; ^3^ MARA Key Laboratory of Surveillance and Management for Plant Quarantine Pests, College of Plant Protection, China Agricultural University, Beijing, China; ^4^ Université Côte d’Azur, INRAE, CNRS, UMR ISA, Nice, France

**Keywords:** resistance evolution, RNA interference, biological traits, lepidoptera, gene expression

## Abstract

Cyantraniliprole, a second-generation anthranilic diamide, is widely used to manage lepidopteran pests, including the invasive tomato pinworm *Tuta absoluta* (Meyrick). However, cyantraniliprole’s resistance mechanisms and associated fitness costs in *T. absoluta* remain underexplored. Here, we investigated the fitness costs and resistance mechanisms of cyantraniliprole-resistant strain (CyanRS) via nanocarrier-mediated RNA interference (RNAi). Results showed that the egg incubation period and developmental durations of larval and pupal stages were significantly prolonged in the CyanRS population of *T. absoluta* compared to the susceptible strain (SS). Further, the adult emergence, longevities of male and female, fecundity, and hatching rate were significantly reduced in CyanRS individuals. The mRNA expression levels of cytochrome b5 reductase (*CYB5R*) and cytochrome P450 (*CYP9A306*) were analyzed using RT-qPCR to explore their potential involvement in cyantraniliprole resistance in *T. absoluta*. Phylogenetic and motif analysis of *CYB5R* and *CYP9A306* indicated their evolutionary and functional conservation with other insect species, especially Lepidopterans. Notably, nanocarrier-encapsulated dsRNA targeting *CYB5R* and *CYP9A306* genes significantly reduced their expression levels. Further, the activity of cytochrome P450 was substantially decreased after the knockdown of the *CYB5R* and *CYP9A306* genes. This increased susceptibility of the resistant population of *T. absoluta* to cyantraniliprole, leading to a higher mortality rate than the controls. These findings show that *CYB5R* and *CYP9A306* might play a key role in cyantraniliprole resistance evolution in *T. absoluta*. The current study provides in-depth insights to understand the underlying mechanisms of cyantraniliprole resistance in this key invasive herbivore.

## Introduction

The South American tomato pinworm, *Tuta absoluta* (Meyrick) (Lepidoptera: Gelechiidae), represents a significant global challenge to agricultural sustainability. Originating from South America, this highly destructive and invasive pest has demonstrated remarkable adaptability, rapidly expanding its geographical range to Europe, Africa, the Middle East, and Asia. Infestations of *T. absoluta* have severely impacted tomato production in both open fields and controlled greenhouse systems, with yield losses reaching up to 80–100% globally (Desneux et al., 2010). Since its first identification in China in 2017, the pest has spread rapidly, infesting over ten provinces within a few years (Zhang et al., 2021). Beyond tomatoes, *T. absoluta* also targets other economically vital solanaceous crops, including potatoes, peppers, eggplants, and tobacco (Desneux et al., 2011; Guillemaud et al., 2015; Biondi et al., 2018). Its aggressive colonization and adaptability have established *T. absoluta* as a critical threat to global agriculture.

One of the primary challenges in managing *T. absoluta* is its extraordinary ability to develop resistance to various classes of insecticides (Silva et al., 2016; Roditakis et al., 2018). The resistance evolution compromises the effectiveness of chemical control measures, often the first line of defense against invasive pests. For instance, *T. absoluta* populations in Brazil and China have exhibited notable resistance to key diamides, imposing the development of insecticides with new modes of action and innovative pest management strategies (Lahm et al., 2007; Huang et al., 2021). Cyantraniliprole, a second-generation anthranilic diamide, has become a crucial insecticide for managing *T. absoluta* and other pests. It targets ryanodine receptors, disrupts calcium ion balance, and causes muscle paralysis and death (Jeanguenat, 2013). While cyantraniliprole exhibits broad-spectrum activity against various insect orders and is particularly effective against both chewing and sucking pests, resistance development has already been documented. However, the potential for rapid resistance development, driven by cross-resistance and heavy usage, highlights the importance of proactive resistance management strategies (Huang et al., 2021; Sun et al., 2023; Sun et al., 2024). Understanding resistance mechanisms and associated fitness costs is critical for designing effective control strategies for *T. absoluta*.

The upregulation of cytochrome P450 enzymes during resistance development in *T. absoluta* leads to decreased population growth rates since it creates substantial fitness costs manifesting when insecticide pressure disappears. By investing metabolic resources in resistance mechanisms, insects suffer fitness costs that trigger reduced fecundity stress during development and growth, impairing total fitness (Tchouakui et al., 2020; Gul et al., 2023a). The population of *Anopheles funestus* stands as one example among other species where *T. absoluta* populations demonstrate identical stress reactions that weaken their numbers (Tchouakui et al., 2020). Under stressful environmental conditions, researchers have found reduced net reproductive rates (*R*_0_) and intrinsic rates of increase (*r*), providing evidence for this concept (Gharekhani et al., 2023). Reducing insecticide usage provides an avenue for integrated pest management (IPM) since fitness costs lead to resistance reversal when susceptible individuals outcompete resistant individuals (Belinato and Martins, 2016; Gassmann, 2023). The evolutionary potential of compensatory mechanisms like modifier genes, which help reduce fitness costs, implies continuous monitoring for effective long-term resistance management systems (Belinato and Martins, 2016; Pang et al., 2021). Further, the cytochrome P450 (*CYP450*) monooxygenases are crucial for insecticide detoxification in insect pests. Cytochrome P450 reductase (CPR) and *CYB5R* play essential roles in electron transfer, which is crucial for *CYP450* activity. Co-expression of CPR significantly enhances detoxification efficiency in insects (Gong et al., 2022; Ibrahim et al., 2024). *CYP9A306* plays a specific role in enhancing detoxification, while *CYB5R* is crucial for electron transfer, highlighting the effectiveness of these pathways (Roditakis et al., 2017; Guo et al., 2024). Previous studies reported the role of several P450 genes such as *CYP339A1*, *CYP340G2*, *CYP6AE70*, and *CYP321A19* in insecticide resistance in different insect species (Hu et al., 2024; Li et al., 2024).

RNA interference (RNAi) is a robust and powerful post-transcriptional gene silencing tool (Fire et al., 1998) widely used to study gene functions in different insect species (Burand and Hunter, 2013). The success of RNAi experiments heavily depends on the delivery mechanism, which plays a crucial role in ensuring efficient gene silencing, especially in lepidopteran insects. Nowadays, nanocarriers have been extensively utilized to improve the stability, precision of targeted delivery, and cellular uptake across a wide range of insect species (Zhang et al., 2010; Yan et al., 2022). Star polycation (SPc) is an economical gene delivery vector featuring a branched polymer structure with multiple positively charged arms that spontaneously bind to dsRNA via electrostatic interactions, effectively shielding the dsRNA from degradation by RNase A (Li et al., 2019; Ma et al., 2022). The enhanced stability of dsRNA when loaded into nanocarriers significantly improves its ability to bind to insect cells, thereby boosting the overall efficiency of RNAi (Yan et al., 2020).

This study aims to investigate the impact of cyantraniliprole resistance development on the overall fitness of *T. absoluta*. RT-qPCR was employed to check the expression level of *CYP9A306* and *CYB5R* genes possibly involved in cyantraniliprole resistance. Additionally, nanocarrier-mediated RNAi was used to silence these two genes to investigate their potential role in developing cyantraniliprole resistance in *T. absoluta*. These findings provide critical insights for formulating effective resistance management strategies against *T. absoluta*.

## Materials and methods

### *Tuta absoluta* strains

The *T. absoluta* initial colony was collected from Yunnan, China, in June 2018. The susceptible strain (SS) was established and maintained on fresh tomato plants without exposure to any insecticide for several generations. The cyantraniliprole-resistant strain (CyanRS) was developed following eight generations of selection to cyantraniliprole under laboratory conditions. The colonies of both strains (SS and CyanRS) of *T. absoluta* were maintained under laboratory conditions with 25 ± 1°C, 16L:8D photoperiod, 60 ± 5% relative humidity.

### Bioassays

Adult *T. absoluta* from SS and CyanRS populations were released on fresh tomato plants for 12 hours to lay eggs. The plants containing eggs were transferred to clean cages and were allowed for egg hatching and larvae development. The approach was employed to ensure all larvae have the same age and stage for the subsequent bioassay experiments. The *T. absoluta* (3rd instar) was collected from SS and CyanRS populations for the bioassays to check the toxicity of cyantraniliprole using the leaf-dip bioassay technique (Ullah et al., 2024). The stock solution of the technical grade cyantraniliprole with 95% of the active ingredient was prepared using analytical grade acetone. Distilled water containing 0.05% Triton X-100 (Sigma) was used to further serially dilute the stock solution for the bioassay experiments. Fresh tomato leaves were cut from tomato plants and dipped in each cyantraniliprole serial dilution separately for 15 s. The treated tomato leaves were allowed to air dry at room temperature for 1-2 hours. The petioles of all treated leaves were wrapped with cotton wool for moisturization. After drying, all leaves were put into the Petri dishes (diameter: 8 cm; height: 1.5 cm) containing filter paper. The distilled water containing 0.05% Triton X-100 (Sigma) was used as the control treatment. Twenty larvae (3rd instar) of *T. absoluta* were used in one replicate, and each concentration of cyantraniliprole had three replicates. The bioassays were performed under laboratory conditions (25 ± 1°C, 60 ± 5% RH, and a 16:8 h light/dark photoperiod). The mortality was checked after 72 h. The *T. absoluta* larvae were noted as dead if unable to show movement when touched.

### Fitness comparison among CyanRS and SS populations of *T. absoluta*

The impact of cyantraniliprole resistance development on the life-history traits, including developmental period, longevities of male and female, fecundity, hatching rate, and adult emergence of the resistant strain (CyanRS) were compared with the susceptible strain (SS) of *T. absoluta*. Eighty and seventy-five newly laid eggs from SS and CyanRS strains of *T. absoluta* were transferred to clean Petri dishes containing fresh tomato leaves, respectively. The eggs were observed daily, and the duration and hatching were recorded for both strains. Thirty newly hatched *T. absoluta* larvae from SS and CyanRS populations were transferred to clean Petri dishes, respectively. Fresh tomato leaves were provided to each Petri dish, and their petioles were wrapped with wet cotton wool to provide moisture. The developmental period of both strains was observed and recorded daily. Thirty SS and CyanRS strains pupae were individually shifted to a glass tube with a 1.5 cm diameter and 8.0 cm height. The developmental period of the pupae from both populations (SS and CyanRS) was observed and recorded. When the adults emerged, thirty males and thirty females from SS and CyanRS strains of *T. absoluta* were paired in a glass tube with 3.0 cm diameter and 20.0 cm height containing fresh tomato leaves and 10% honey. The longevity and fecundity of both strains were recorded daily. All the fitness comparison experiments were conducted under laboratory conditions with 25 ± 1°C, 16L:8D photoperiod, 60 ± 5% relative humidity.

### RT-qPCR

The mRNA expression levels of *CYP9A306* and *CYB5R* were analyzed using RT-qPCR to explore their potential involvement in cyantraniliprole resistance in *T. absoluta*. Total RNA was extracted from the SS and CyanRS populations of *T. absoluta* (3^rd^ instar) using the RNAsimple Total RNA kit following the recommended protocol. The RNA quality and quantity were determined by the Bioanalyzer Agilent 2100 (Agilent Technologies, USA). 1μg of total RNA was used to synthesize the cDNA using the iScriptTM cDNA Synthesis Kit (Bio-Rad, CA, USA) according to recommended instructions. RT-qPCR was conducted on a 10 μL total volume of a reaction consisting of 5 μL 2^X^ Kappa SYBR Green I qPCR Mix, 0.2 μL forward and reverse primers (10 μM each), 1 μL of cDNA template, and the remaining volume was nuclease-free water using a CFX Connect TM Real-Time System (Bio-Rad, United States). The thermocycling conditions of each qPCR consist of 95°C for 45 sec, followed by 40 cycles of 95°C for 15 sec, 50–65°C for 15 sec, and 70°C for 30-60 sec. Gene expressions were calculated using 2^-ΔΔCt^ method (Livak and Schmittgen, 2001). Elongation factor 1 alpha (*EF1α*) and ribosomal protein L28 (*RPL28*) were used as housekeeping genes to normalize the gene expressions. RT-qPCR experiments consist of three biological and three technical replicates. The primers used in the current study are presented in **Table 1**.

**Table 1:** Primer sequences used for RT-qPCR and dsRNA synthesis.

Primer name	Forward sequence	Reverse sequence
*CYP9A306*	CGAGGTGAAAATCATGGCGT	CAGTGTCCACCCTTCATCCT
*CYB5R*	CGAGAGCGGGAAAATTGAGG	CGACTGTTCTTGGTGACGTC
*RPL28*	TCAGACGTGCTGAACACACA	GCCAGTCTTGGACAACCATT
*TaEF1α*	GAAGCCTGGTATGGTTGTCGT	GGGTGGGTTGTTCTTTGTG
*dsEGFP*	TAATACGACTCACTATAGGGAAGTTCAGCGTGTCCGGCGAGG	TAATACGACTCACTATAGGGCACCTTGATGCCGTTCTTCTGC
ds*CYP9A306*	taatacgactcactatagggTCCTTCTTCACGAGTTGGCT	taatacgactcactatagggACGTTGAAGGTGGAGGTGTC
ds*CYB5R*	taatacgactcactatagggTCGTGTAGTGAGCAAATCGC	taatacgactcactatagggTGTCGTCTTCTTTCGCAATG

### Phylogenetic analysis

A nucleotides blast (blastn) search of the National Center for Biotechnology Information (NCBI) (https://blast.ncbi.nlm.nih.gov/Blast.cgi) was used to download the amino acid sequence of homologous genes of cytochrome b5 reductase (*CYB5R*) and cytochrome P450 (*CYP9A306*) of different insect species. These homologous genes were selected based on the degree of homology: 96%, 95%, and 90%, respectively. Therefore, to elucidate the evolutionary relationship of *T. absoluta CYB5R* and *CYP9A306*, an analysis of the phylogenetic tree using the full-length amino acid sequences was made on orthologs belonging to different species of insects. Complete amino acid sequences of *CYB5R* and *CYP9A306* orthologs were aligned using ClustalW with MEGA11. The phylogenetic trees were reconstructed based on the maximum likelihood (ML) approach with the *p*-distance model of amino acid substitution. Missing data treatment as “available case” or “pairwise deletion” for gaps/missing data and have selected 1000 bootstrap replications (Tamura et al., 2011). The species and their gene name of the *CYB5R* and *CYP9A306* genes used to generate the tree were given in (**Supplementary Table S1**). The MEME 5.5.7 database identified all the motifs with the complete amino acid sequences of *CYP9A306* and *CYB5R*. Lengths of each *CYP9A306* and *CYB5R* motif were demonstrated proportionally.

### Synthesis of double-stranded RNA and dsRNA/SPc nanoparticle complex

The dsRNAs i.e., ds*CYB5R*, ds*CYP9A306*, and ds*EGFP*, were synthesized using 491, 532, and 413 bp PCR products of targeted genes for, respectively, by T7 RNAi Transcription Kit (Nanjing Vazyme Biotech Co.,Ltd. China). To amplify the targeted genes dsRNA, a T7 promoter (TAATACGACTCACTATAGGG) was attached to the 5’ end of the primers. The PCR reaction includes 8 μL of NTP Mix, 2 μL of 10 × Transcription Buffer, 2 μL of T7 Enzyme Mix, and 8 μL of DNA Template per tube. The samples were incubated for 2 h at 37°C. The 20 μL of transcription product, 17 μL of RNase-free H_2_O, 2 μL of RNase T1, and 1 μL of DNase I were used to prepare the double enzymes digestion system. The tubes were mixed and put in the PCR at 30°C for 30 min. After purification, the dsRNA was dissolved using RNase free H_2_O. The quality and quantity of dsRNA were checked by Quawell UV-Vis Q5000 spectrophotometer (Quawell Technology Inc., San Jose, CA, USA). The double-stranded enhanced green fluorescent protein (ds*EGFP*) was used as a control. The Star polycation (SPc), presented by China Agricultural University, was mixed with the dsRNA with a 1:1 mass ratio. The final concentrations for SPc and dsRNA were 500 ng μL^−1^. The dsRNA/SPc complex was prepared after 15 min incubation at room temperature (Yan et al., 2022; Ma et al., 2023). The primers used in the current study are shown in **Table 1**. The prepared dsRNA/SPc complex samples were stored at -20°C until further experiments.

### Nanocarrier-mediated RNA interference and bioassays

Complexes of ds*CYB5R*/SPc, ds*CYP9A306*/SPc, and ds*EGFP*/SPc with a final concentration of 500 ng μL^−1^ were evenly sprayed on fresh tomato leaves. Wet cotton wool was wrapped to the petiole of each leaf for moisturization and was allowed to dry at room temperature for about 1-2 hours. The treated leaves were shifted to clean Petri dishes containing filter paper. All experiments have three replications. Twenty larvae of *T. absoluta* (2nd instar) from CyanRS were used per replicate. ds*EGFP*/SPc and DEPC-water were used as controls. After 48 h feeding on dsRNA/SPc complexes and DEPC-water, the larvae were collected for P450 enzyme activity checking and RNA extraction as mentioned above. RT-qPCR and enzyme activity were carried out to check the silencing efficiency of P450 genes. All experiments were conducted under laboratory conditions (25 ± 1°C, 60 ± 5% RH, and a 16:8 h light/dark photoperiod).

Additionally, the sensitivity of the resistant strain of *T. absoluta* (CyanRS) to cyantraniliprole was checked via a leaf-dip bioassays approach. The 3rd instar *T. absoluta* larvae were transferred to tomato leaves treated with LC_50_ (17.27 mg/L) of cyantraniliprole to CyanRS following 48 h feeding on ds*CYB5R*/SPc, ds*CYP9A306*/SPc, ds*EGFP*/SPc and DEPC-water. The larvae fed on ds*EGFP*/SPc and DEPC water were considered as control groups. The mortality was checked after 72 h of exposure and the larvae were considered dead if unable to show movement when touched. Each bioassay consists of three replicates, and all experiments were conducted under laboratory conditions with 25 ± 1°C, 60 ± 5% RH, and a 16:8 h light/dark photoperiod.

### Data analysis

POLO Plus 2.0 (LeOra Software, CA, USA) was used to analyze the bioassay data via log-probit model. The fitness data, RT-qPCR, enzyme activity, and mortality were analyzed by Student’s t-test and one-way analysis of variance (ANOVA) with Tukey’s *post hoc* test via SPSS version 29 (SPSS Inc., IL, USA). For all experiments, *P* < 0.05 was considered significant. The figures were constructed with GraphPad Prism 9 (GraphPad Software, MA, USA) and BioRender.com.

## Results

### Toxicity of cyantraniliprole against *T. absoluta*

Cyantraniliprole-resistant strain (CyanRS) of *T. absoluta* was previously developed from laboratory susceptible strain (SS) following eight generations of selection. Bioassay results indicated that the LC_50_ of SS *T. absoluta* was 0.72 mg L^-1^ (0.60-0.85 mg L^-1^) with Slope ± SE of 2.084 ± 0.185, χ^2^ = 9.341, df = 16, and *P* = 0.899. The LC_50_ of CyanRS population of *T. absoluta* was 17.30 mg L^-1^ (14.00-22.42 mg L^-1^) with Slope ± SE of 1.897 ± 0.203, χ^2^ = 7.199, df = 16, and *P* = 0.969. The resistant strain of *T. absoluta* (CyanRS) developed 23.9-fold resistance against cyantraniliprole compared to the susceptible population.

### Impact of cyantraniliprole resistance on the fitness of *T. absoluta*

**Figure 1:**
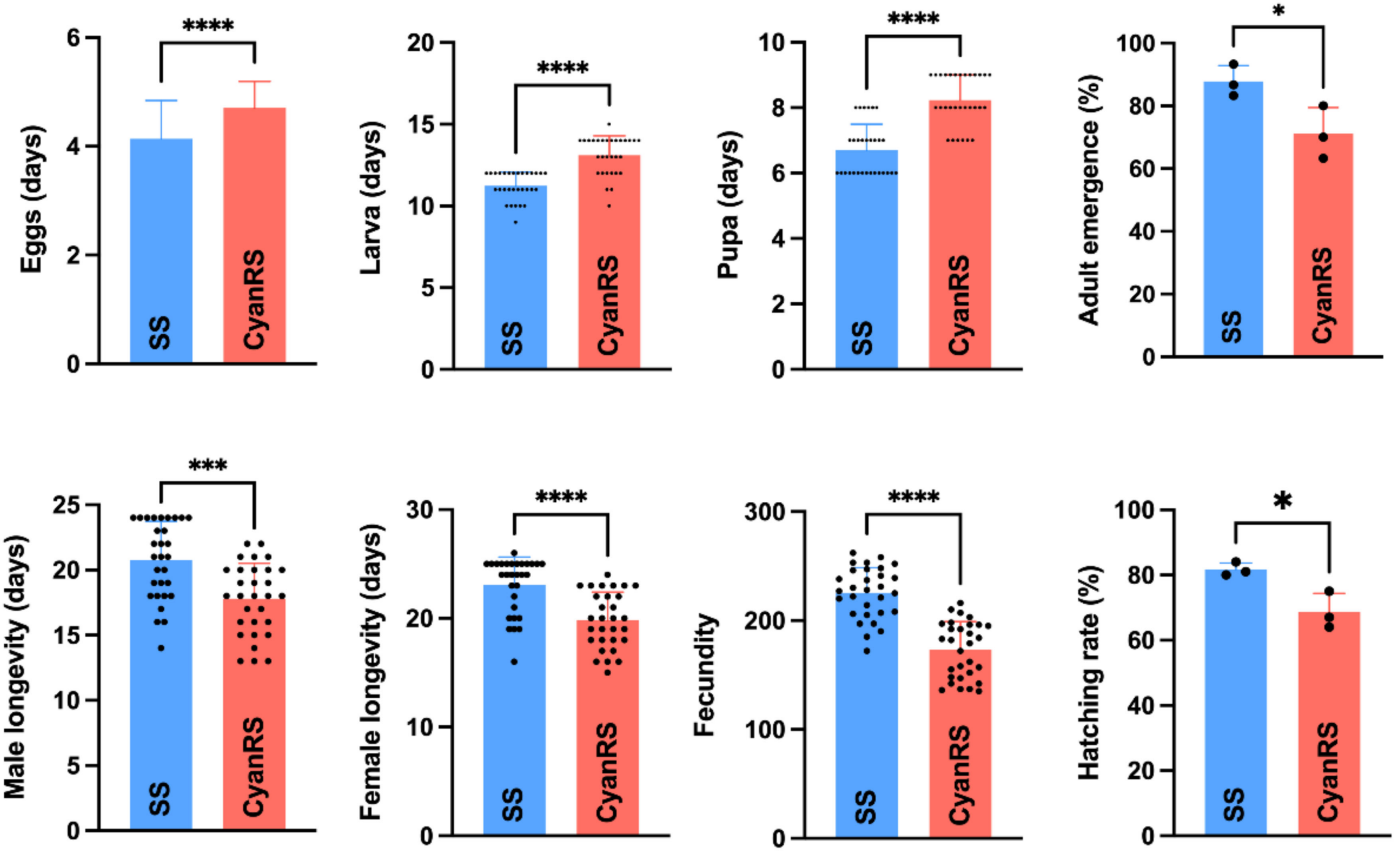
Effects of cyantraniliprole resistance on the overall fitness, including eggs, larvae, pupae, adult emergence, male and female longevity, fecundity, and hatching rates of susceptible (SS) and resistant strains (CyanRS) of *Tuta absoluta*. Data presented as mean ± SE and the asterisks *, ***, and **** show significant differences at *P* < 0.05, *P* < 0.001, and *P* < 0.0001 based on Student’s t-test.

The impact of cyantraniliprole resistance development on the overall fitness of *T. absoluta* is shown in **Figure 1**. Results showed significantly prolonged egg incubation time (*t*-test: *t* = 5.80, df = 153, *P* < 0.0001), larvae period (*t*-test: *t* = 6.989, df = 58, *P* < 0.0001), and pupal developmental duration (*t*-test: *t* = 7.573, df = 58, *P* < 0.0001) in the cyantraniliprole-resistant *T. absoluta* as compared to susceptible strain (**Figure 1**). Additionally, the male (*t*-test: *t* = 4.044, df = 58, *P* = 0.0002) and female (*t*-test: *t* = 4.858, df = 58, *P* < 0.0001) longevities of the CyanRS population of *T. absoluta* were significantly decreased compared to SS individuals. In the CyanRS strain, the fecundity (*t*-test: *t* = 8.193, df = 58, *P* < 0.0001), hatching rate (*t*-test: *t* = 3.719, df = 4, *P* = 0.02), and adult emergences (*t*-test: *t* = 2.942, df = 4, *P* = 0.04) were substantially lower as compared to the susceptible *T. absoluta* population (**Figure 1**).

### Expression profile of cyantraniliprole resistance-related genes

**Figure 2:**
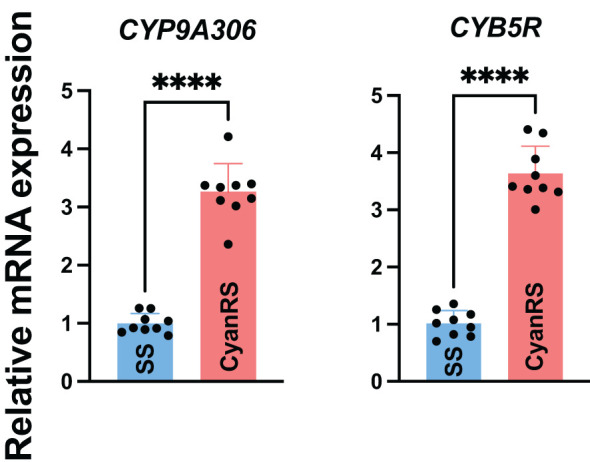
Relative expression levels of *CYP9A306* and *CYB5R* genes in cyantraniliprole-resistant (CyanRS) and susceptible (SS) strains of *Tuta absoluta*. Data presented as mean ± SE of the three independent biological replicates. The asterisks **** show significant differences at *P* < 0.0001, based on Student’s t-test.

Gene expressions of *CYP9A306* and *CYB5R* in response to cyantraniliprole resistance were checked in the resistant (CyanRS) and susceptible strains (SS) of *T. absoluta* via RT-qPCR (**Figure 2**). qPCR results showed that the expression level of *CYP9A306* was significantly (*t-*test: *t* = 13.28, df = 16, *P* < 0.0001) increased 3.260-fold in CyanRS *T. absoluta* compared to the SS population. Similarly, the mRNA expression level of *CYB5R* was substantially (*t-*test: *t* = 14.85, df = 16, *P* < 0.0001) increased by 3.636-fold in resistant *T. absoluta* (CyanRS) as compared to SS individuals (**Figure 2**).

### Phylogenetic analysis

**Figure 3:**
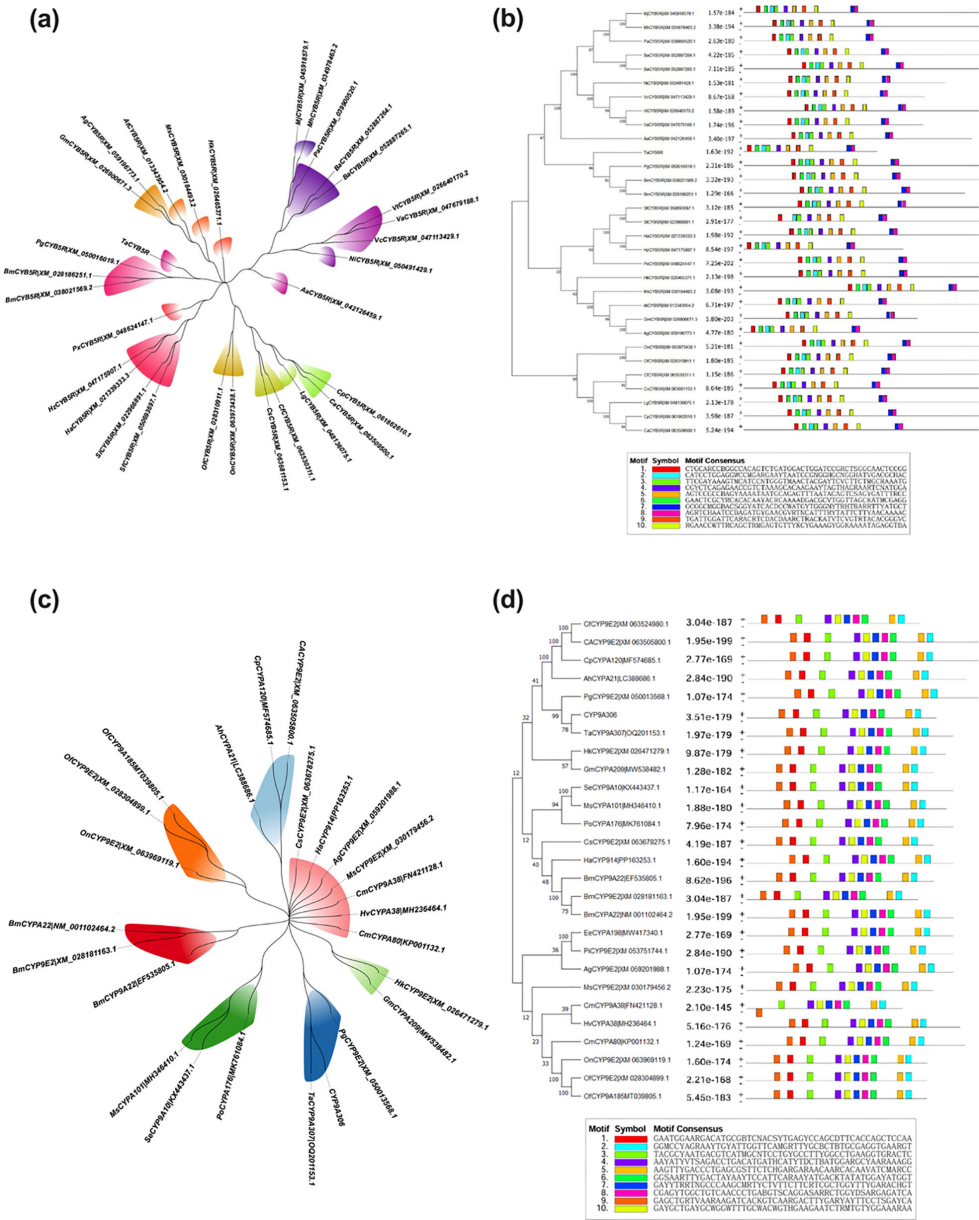
Phylogenetic and motif analysis of the *CYB5R* **(a, b)** and *CYP9A306* **(c, d)** in lepidopteran insect species.

The phylogenetic tree of *CYB5R* indicated that the evolution proximity between species (distinct color-coded in clades), clustering *T. absoluta* close to other Lepidoptera species such as *Bombyx mori* and *Helicoverpa armigera* where it reflects shared ancestry and high sequence similarity as shown in (**Figure 3a**). Such diversification has also occurred at a more distant clade level, as is apparent from the presence of greater divergence (i.e., genetic divergence) between distant clades, such as those containing the *Pectinophora gossypiella* and the *Plutella xylostella*. These phylogenetic relationships are also supported by motif analysis that identifies conserved motifs among species in the same clades. Similar motif patterns of closely related species suggest conserved functional domains central to the gene’s function in metabolic processes. In (**Figure 3b**), motif composition varies between distantly related species to indicate evolutionary divergence and adaptation to different ecological niches.

Phylogenetic and motif analysis of *CYP9A306* elucidate the evolutionary and functional conservation of this gene across insect species. Phylogenetic results indicated that *T. absoluta* (OQ201153.1) clustered closely with Lepidoptera species like *Bombyx mori* (EF535805.1 and NM_001102464.2 and *Helicoverpa armigera* (PP163253.1), and shared ancestry and significant sequence conservation among species as seen in (**Figure 3c**). Genetic divergence beyond the threshold value, greater than 0.07, reveals adaptive evolution caused by ecological or metabolic pressures to distant clades, including *Cydia fagiglandana* (XM_063524980.1), *Ostrinia furnacalis* (XM_028304899.1), and *Plecoptera oculata* (MK761084.1). Similarly, motif arrangements are distinct in species in more divergent clades, as shown in (**Figure 3d**) and point to functional specialization and adaptation. The alignment of conserved motifs to phylogenetic proximity illustrates evolutionary relatedness and preservation of function, whereas the reorganization of motif architecture in distant taxa reflects the genes capacity to adapt to different environmental contexts shown in (**Figure 3d**).

### Nanocarrier-mediated knockdown of P450 genes increases the sensitivity of *T. absoluta* to cyantraniliprole

**Figure 4:**
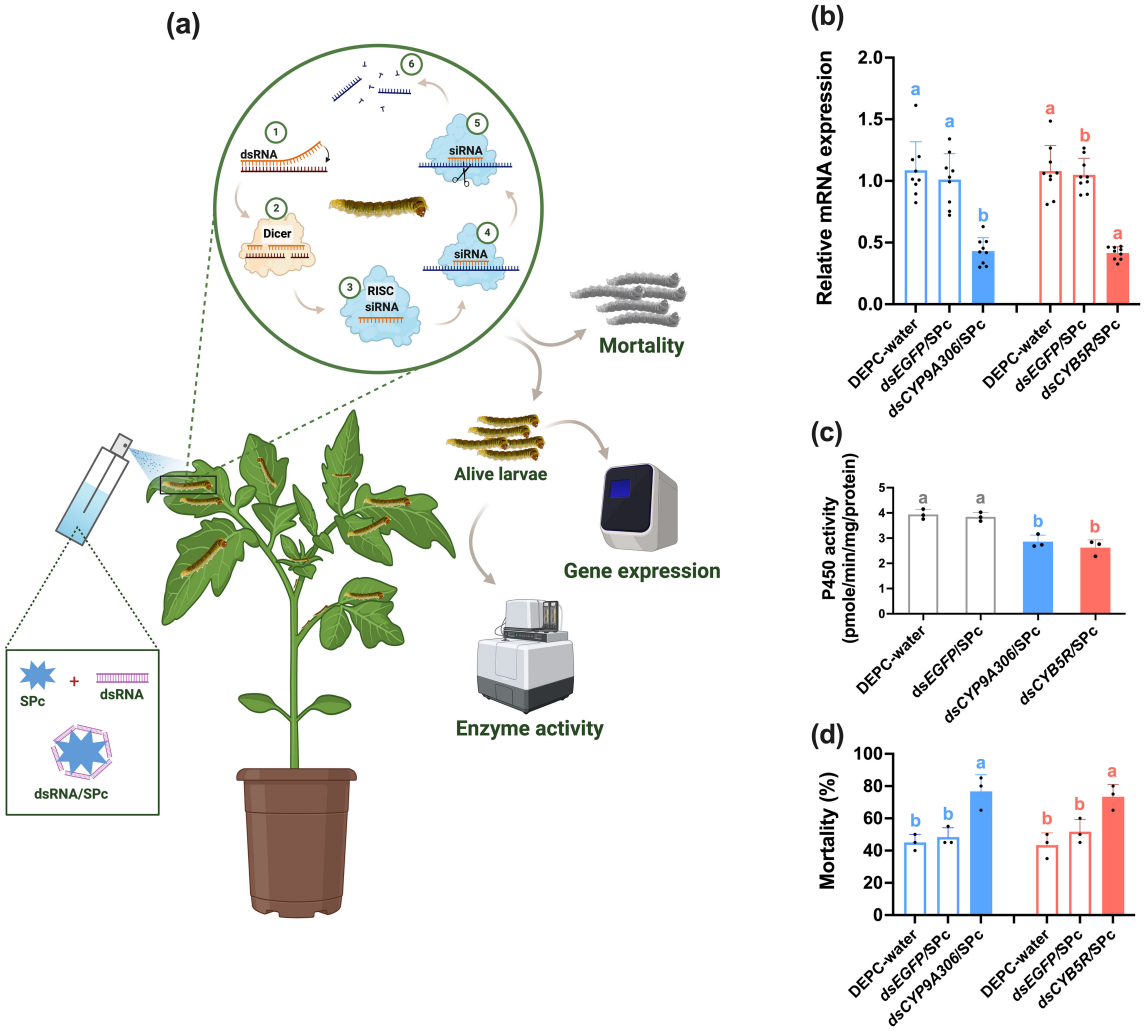
Nanocarrier-mediated RNAi of *CYP9A306* and *CYB5R* genes increase the sensitivity of the cyantraniliprole-resistant strain (CyanRS) of *Tuta absoluta* against cyantraniliprole. **(a)** Schematic diagram of nanocarrier-mediated RNA inference. **(b)** Relative mRNA expression level of *CYP9A306* and *CYB5R* genes in CyanRS and SS populations of *Tuta absoluta*. Data presented as mean ± SE of the three independent biological replicates. **(c)** The activity of cytochrome P450 enzyme among CyanRS and SS populations of *Tuta absoluta*. **(d)** Mortality rates (%) of cyantraniliprole-resistant strain of *Tuta absoluta* at 48 h of feeding on ds*CYP9A306*/SPc, ds*CYB5R*/SPc, ds*EGFP*/SPc and DEPC water after exposure to the LC_50_ of cyantraniliprole. Different lowercase letters represent significant differences at *P*<0.001 level (one-way analysis of variance (ANOVA) with Tukey’s post hoc test.

RT-qPCR showed the overexpression of *CYP9A306* and *CYB5R* genes in response to cyantraniliprole resistance in *T. absoluta* compared to susceptible population. For the functional validation, a nanocarrier-mediated RNAi approach was employed to knockdown *CYP9A306* and *CYB5R* genes in the resistant strain (CyanRS) of *T. absoluta* (**Figure 4a**). The RNAi efficiency of these targeted genes was checked via RT-qPCR after feeding CyanRS *T. absoluta* on ds*CYB5R*/SPc and ds*CYP9A306*/SPc for 48 h as compared to ds*EGFP*/SPc and DEPC-water. RT-qPCR results indicated that the expression level of *CYP9A306* was significantly decreased (one-way ANOVA, Tukey’s HSD test, *F*_2,26_ = 31.621, *P* < 0.001) with 0.43-fold (57%) as compared to ds*EGFP*/SPc and DEPC water (**Figure 4b**). Similarly, the mRNA expression level of *CYB5R* was dramatically reduced (one-way ANOVA, Tukey’s HSD test, *F*_2,26_ = 59.479, *P* < 0.001) by 59% (0.41-fold) compared to ds*EGFP*/SPc and DEPC water treated *T. absoluta*. After feeding ds*CYP9A306*/SPc and ds*CYB5R*/SPc, the cytochrome P450 enzyme activity was significantly (one-way ANOVA, Tukey’s HSD test, *F*_3,11_ = 23.461, *P* < 0.001) reduced in the CyanRS population of *T. absoluta* as compared to controls (ds*EGFP*/SPc and DEPC-water) (**Figure 4c**). Results showed that the cytochrome P450 enzyme activity was around 26.92% decreased when CyanRS individuals of *T. absoluta* was fed on ds*CYP9A306*/SPc as compared to ds*EGFP*/SPc and DEPC water (**Figure 4c**). Similarly, the P450 enzyme activity was reduced by 32.82% following feeding on ds*CYB5R*/SPc compared to controls (ds*EGFP*/SPc and DEPC water). To check the sensitivity of resistant *T. absoluta* to cyantraniliprole, the *T. absoluta* larvae (3rd instar) were exposed to the LC_50_ value of CyanRS (0.72 mg L^-1^) for 72 h following feeding on ds*CYP9A306*/SPc, ds*CYB5R*/SPc, ds*EGFP*/SPc and DEPC water. Compared to controls, the sensitivity of the CyanRS population of *T. absoluta* was substantially increased following the nanocarrier-mediated knockdown of *CYP9A306* and *CYB5R* genes. The bioassay results indicated significantly increased mortalities of 76.6% (one-way ANOVA, Tukey’s HSD test, *F*_2,8_ = 16.350, *P* = 0.004) and 73.3% (one-way ANOVA, Tukey’s HSD test, *F*_2,8_ = 12.333, *P* = 0.007) in the ds*CYP9A306*/SPc and ds*CYB5R*/SPc exposed *T. absoluta*, respectively, as compared to ds*EGFP*/SPc and DEPC water (**Figure 4d**).

## Discussion

Our study provides compelling evidence for the development of cyantraniliprole resistance in *T. absoluta*. The resistant strain (CyanRS) exhibited a 23.9-fold increase in LC_50_ compared to the susceptible strain (SS). This resistance imposes significant fitness costs on *T. absoluta*, including prolonged egg incubation, larval, and pupal developmental periods, as well as reduced fecundity, hatching rates, and adult emergence. Gene expression analysis revealed a marked upregulation of *CYP9A306* and *CYB5R* in the CyanRS strain. By utilizing nanocarrier-mediated RNAi to knockdown these genes, we observed reduced gene expression, decreased cytochrome P450 enzyme activity, and increased mortality when exposed to the LC_50_ of cyantraniliprole. These findings collectively highlight the critical role of *CYP9A306* and *CYB5R* in cyantraniliprole resistance and suggest that targeting these genes via RNAi could serve as a promising strategy for managing resistance in *T. absoluta* populations.

Cyantraniliprole resistance imposes significant fitness costs related to delayed developmental stages, reduced adult longevity, and impaired reproductive attributes, indicating a trade-off between insecticide survival and physiological performance. These findings are consistent with our recent study in which moderate spinosad resistance induces significant fitness costs by negatively affecting the development and reproduction of the resistant population of *T. absoluta* (Ullah et al., 2025). As demonstrated in other lepidopteran species, *T. absoluta* also exhibited resistance-related nutrient utilization and energy efficiency reductions (Xu et al., 2016). Our findings are in accord with general features of metabolic costs of detoxification enzyme activity and associated genetic mutations that lead to physiological impairments, slower development as well as reduced reproduction in other resistant insect species (Kliot and Ghanim, 2012; Kang et al., 2017; Gul et al., 2023a). We also found that the fitness costs resulted in resistance reversal following reduced selection pressure, consistent with the results found in spinosad-resistant *T. absoluta* populations (Campos et al., 2014). Similar patterns have been observed in other lepidopteran pests, such as reduced survival and reproduction in natural conditions (Nozad-Bonab et al., 2021). Research across various insect species supports these findings, indicating slower development rates and impaired locomotion in resistant strains (Freeman et al., 2021; Zeng et al., 2022; Gul et al., 2023b). *λ*-cyhalothrin-resistant population of *Cydia pomonella* exhibited fitness costs, including delays in development, reduced fecundity, and slower population growth (Hu et al., 2024). Furthermore, its efficacy in reducing the survival and reproduction of *T. absoluta* makes it a very good candidate for use as a key control measure for this pest while highlighting the need for sustainable approaches to delay resistance development (Martins et al., 2024).

Cytochrome P450 (*CYP450*) monooxygenases are major insecticide detoxication enzymes implicated in insecticide resistance in many species. In this study, overexpression of *CYP9A306* and *CYB5R* was observed in the CyanRS strain compared to SS, consistent with previous research on *T. absoluta* (Stavrakaki et al., 2022; Bavithra et al., 2024). Cytochrome P450 reductase (*CPR*) and *CYB5R* facilitate electron transfer and, together with CPR co-expression, are critical for *CYP450* activity and an increase in detoxification efficiency in *Anopheles funestus* and *Culex quinquefasciatus* (Gong et al., 2022; Ibrahim et al., 2024). In addition, specific roles for *CYP9A306* in enhanced detoxification and for *CYB5R* in electron transfer further underscore the potency of these pathways (Roditakis et al., 2017; Guo et al., 2024). Li et al. reported that RNAi-mediated silencing of *CYP339A1*, *CYP340G2*, and *CYP321A19* significantly increased the sensitivity of resistant strains of *Spodoptera litura* to indoxacarb (Li et al., 2024). The overexpression of the *CYP6AE70* confers resistance to multiple insecticides in *Spodoptera exigua* (Hu et al., 2024). Ullah et al. (2023) reported the functional importance of these P450 genes in insecticide resistance development and revealed that overexpression of *CYP6CY14* and *CYP6DC1* genes linked with clothianidin resistance in melon aphids (Ullah et al., 2023). Overall, these findings reinforce the versatility and broad relevance of P450 enzymes in pest resistance management strategies.

Nanocarrier-mediated RNAi targeting these genes effectively reduced their expression, leading to significantly increased mortality, underscoring their critical role in cyantraniliprole resistance. Based on previous studies highlighting the stability and efficacy of nanocarrier-bound dsRNA in pest control (Yan et al., 2022; Chao et al., 2023; Wang et al., 2023), our findings validate this technology’s practical potential, especially in determining the resistance mechanisms of commonly used insecticides. The successful application of nanocarrier-mediated RNAi highlights its potential as an effective tool for integrated pest management. By addressing challenges such as dsRNA degradation and low uptake efficiency, the SPc complex enhances delivery and stability. Previous studies have demonstrated RNAi’s effectiveness in pest control (Edwards et al., 2020; Ma et al., 2024) and our findings further support its viability for agricultural applications, specifically for the functional characterization of key genes linked with insecticide resistance. The spray application of dsRNA/SPc complexes targeting *CYP9A306* and *CYB5R* significantly increased the sensitivity of the resistant population of *T. absoluta*, indicating their possible role in cyantraniliprole resistance development. Future research should prioritize field trials to evaluate nanocarrier-mediated RNAi under diverse agricultural conditions, especially for determining resistance mechanisms in the field-collected resistant population of insect pests. Expanding RNAi targets to include additional resistance-related genes and pathways may provide a comprehensive solution for managing resistant population of targeted insect pests.

## Conclusion

To conclude, the current study indicated a tradeoff among resistance degree and fitness costs in the cyantraniliprole-resistant strain of *T. absoluta* compared to the susceptible population. Furthermore, the overexpression of *CYB5R* and *CYP9A306* might have a key role in cyantraniliprole resistance evolution. Additionally, the nanocarrier-mediated silencing of *CYB5R* and *CYP9A306* significantly reduced their mRNA expressions as well as the activity of cytochrome P450 enzyme ultimately increases the susceptibility of the CyanRS population of *T. absoluta* to cyantraniliprole. Overall, these findings provide in-depth information to understand the cyantraniliprole resistance mechanisms, which might be crucial for the rational resistance management of this key invasive herbivore.

## Data Availability

The original contributions presented in the study are included in the article/**Supplementary Material**. Further inquiries can be directed to the corresponding authors.

## References

[B1] Bavithra C. M. M. L. Murugan M. Balasubramani V. Harish S. Prakash K. (2024). Baseline susceptibility of an A1 quarantine pest-the South American tomato pinworm *Tuta absoluta* (Lepidoptera: Gelechiidae) to insecticides: past incidents and future probabilities in line to implementing successful pest management. Front. Plant Sci. 15, 1404250. doi: 10.3389/fpls.2024.1404250 39286840 PMC11404364

[B2] Belinato T. A. Martins A. J. (2016). Insecticide resistance and fitness cost. Insecticides Resistance, 243–261. doi: 10.5772/61826

[B3] Biondi A. Guedes R. N. C. Wan F.-H. Desneux N. (2018). Ecology, worldwide spread, and management of the invasive South American tomato pinworm, *Tuta absoluta*: past, present, and future. Annu. Rev. Entomology 63, 239–258. doi: 10.1146/annurev-ento-031616-034933 28977774

[B4] Burand J. P. Hunter W. B. (2013). RNAi: future in insect management. J. Invertebrate Pathol. 112, S68–S74. doi: 10.1016/j.jip.2012.07.012 22841639

[B5] Campos M. R. Rodrigues A. R. S. Silva W. M. Silva T. B. M. Silva V. R. F. Guedes R. N. C.. (2014). Spinosad and the tomato borer Tuta absoluta: a bioinsecticide, an invasive pest threat, and high insecticide resistance. PloS One 9, e103235. doi: 10.1371/journal.pone.0103235 25122089 PMC4133407

[B6] Chao Z. Ma Z. Zhang Y. Yan S. Shen J. (2023). Establishment of star polycation-based RNA interference system in all developmental stages of fall armyworm Spodoptera frugiperda. Entomologia Generalis 43, 127–137. doi: 10.1127/entomologia/2023/1906

[B7] Desneux N. Luna M. G. Guillemaud T. Urbaneja A. (2011). The invasive South American tomato pinworm, *Tuta absoluta*, continues to spread in Afro-Eurasia and beyond: the new threat to tomato world production. J. Pest Sci. 84, 403–408. doi: 10.1007/s10340-011-0398-6

[B8] Desneux N. Wajnberg E. Wyckhuys K. A. Burgio G. Arpaia S. Narváez-Vasquez C. A.. (2010). Biological invasion of European tomato crops by *Tuta absoluta*: ecology, geographic expansion and prospects for biological control. J. Pest Sci. 83, 197–215. doi: 10.1007/s10340-010-0321-6

[B9] Edwards C. H. Christie C. R. Masotti A. Celluzzi A. Caporali A. Campbell E. M. (2020). Dendrimer-coated carbon nanotubes deliver dsRNA and increase the efficacy of gene knockdown in the red flour beetle Tribolium castaneum. Sci. Rep. 10, 12422. doi: 10.1038/s41598-020-69068-x 32709999 PMC7381663

[B10] Fire A. Xu S. Montgomery M. K. Kostas S. A. Driver S. E. Mello C. C. (1998). Potent and specific genetic interference by double-stranded RNA in Caenorhabditis elegans. Nature 391, 806. doi: 10.1038/35888 9486653

[B11] Freeman J. C. Smith L. B. Silva J. J. Fan Y. Sun H. Scott J. G. (2021). Fitness studies of insecticide resistant strains: lessons learned and future directions. Pest Manage. Sci. 77, 3847–3856. doi: 10.1002/ps.v77.9 33506993

[B12] Gassmann A. J. (2023). “Fitness costs of resistance and their potential application for insect resistance management,” in Insect Resistance Management (Elsevier), 465–491. doi: 10.1016/b978-0-12-823787-8.00004-0

[B13] Gharekhani G. Salekebrahimi H. Chi H. (2023). Demography of *Tuta absoluta* (Meyrick)(Lepidoptera: Gelechiidae) reared on elicitor-treated tomato plants with an innovative comparison of projected population sizes and application of the multinomial theorem for population survival. Pest Manage. Sci. 79, 4964–4976. doi: 10.1002/ps.v79.12 37535824

[B14] Gong Y. Li T. Li Q. Liu S. Liu N. (2022). The central role of multiple P450 genes and their co-factor CPR in the development of permethrin resistance in the mosquito Culex quinquefasciatus. Front. Physiol. 12, 802584. doi: 10.3389/fphys.2021.802584 35095564 PMC8792746

[B15] Guillemaud T. Blin A. Le Goff I. Desneux N. Reyes M. Tabone E.. (2015). The tomato borer, *Tuta absoluta*, invading the Mediterranean Basin, originates from a single introduction from Central Chile. Sci. Rep. 5, 8371. doi: 10.1038/srep08371 25667134 PMC4322357

[B16] Gul H. Gadratagi B. G. Güncan A. Tyagi S. Ullah F. Desneux N.. (2023a). Fitness costs of resistance to insecticides in insects. Front. Physiol. 14, 1238111. doi: 10.3389/fphys.2023.1238111 37929209 PMC10620942

[B17] Gul H. Ullah F. Güncan A. Desneux N. Liu X. (2023b). Thiamethoxam, bifenthrin, and flonicamid resistance in *Schizaphis graminum* and associated fitness costs. Entomologia Generalis 43, 575–586. doi: 10.1127/entomologia/2023/2010

[B18] Guo Y.-A. Si F.-L. Han B.-Z. Qiao L. Chen B. (2024). Identification and functional validation of P450 genes associated with pyrethroid resistance in the malaria vector *Anopheles sinensis* (Diptera Culicidae). Acta Tropica 260, 107413. doi: 10.1016/j.actatropica.2024.107413 39343287

[B19] Hu B. Xing Z. Dong H. Chen X. Ren M. Liu K.. (2024). Cytochrome P450 *CYP6AE70* confers resistance to multiple insecticides in a lepidopteran pest, spodoptera exigua. J. Agric. Food Chem. 72, 23141–23150. 39382406 10.1021/acs.jafc.4c04872

[B20] Hu C. Zhang C. Tang Y.-F. Liu Y.-X. Xia Z.-N. Wang Y.. (2024). Stability, inheritance, cross-resistance, and fitness cost of resistance to λ-cyhalothrin in cydia pomonella. J. Agric. Food Chem. 72, 23520–23532. doi: 10.1021/acs.jafc.4c07166 39385681

[B21] Huang J.-M. Zhao Y.-X. Sun H. Ni H. Liu C. Wang X.. (2021). Monitoring and mechanisms of insecticide resistance in *Spodoptera exigua* (Lepidoptera: Noctuidae), with special reference to diamides. Pesticide Biochem. Physiol. 174, 104831. doi: 10.1016/j.pestbp.2021.104831 33838702

[B22] Ibrahim S. S. Kouamo M. F. Muhammad A. Irving H. Riveron J. M. Tchouakui M.. (2024). Functional validation of endogenous redox partner cytochrome P450 reductase reveals the key P450s CYP6P9a/-b as broad substrate metabolizers conferring cross-resistance to different insecticide classes in Anopheles funestus. Int. J. Mol. Sci. 25, 8092. doi: 10.3390/ijms25158092 39125661 PMC11311542

[B23] Jeanguenat A. (2013). The story of a new insecticidal chemistry class: the diamides. Pest Manage. Sci. 69, 7–14. doi: 10.1002/ps.3406 23034936

[B24] Kang W. J. Koo H. N. Jeong D. H. Kim H. K. Kim J. Kim G. H. (2017). Functional and genetic characteristics of chlorantraniliprole resistance in the diamondback moth, *Plutella xylostella* (Lepidoptera: Plutellidae). Entomological Res. 47, 394–403. doi: 10.1111/1748-5967.12258

[B25] Kliot A. Ghanim M. (2012). Fitness costs associated with insecticide resistance. Pest Manage. Sci. 68, 1431–1437. doi: 10.1002/ps.3395 22945853

[B26] Lahm G. P. Stevenson T. M. Selby T. P. Freudenberger J. H. Cordova D. Flexner L.. (2007). Rynaxypyr™: a new insecticidal anthranilic diamide that acts as a potent and selective ryanodine receptor activator. Bioorganic Medicinal Chem. Lett. 17, 6274–6279. doi: 10.1016/j.bmcl.2007.09.012 17884492

[B27] Li J. Qian J. Xu Y. Yan S. Shen J. Yin M. (2019). A facile-synthesized star polycation constructed as a highly efficient gene vector in pest management. ACS Sustain. Chem. Eng. 7, 6316–6322. doi: 10.1021/acssuschemeng.9b00004

[B28] Li W. Yang W. Shi Y. Yang X. Liu S. Liao X.. (2024). Comprehensive analysis of the overexpressed cytochrome P450-based insecticide resistance mechanism in Spodoptera litura. J. Hazardous Materials 461, 132605. 10.1016/j.jhazmat.2023.132605 10.1016/j.jhazmat.2023.132605 37748309

[B29] Livak K. J. Schmittgen T. D. (2001). Analysis of relative gene expression data using real-time quantitative PCR and the 2– ΔΔCT method. Methods 25, 402–408. doi: 10.1006/meth.2001.1262 11846609

[B30] Ma Z. Zhang Y. Li M. Chao Z. Du X. Yan S.. (2023). A first greenhouse application of bacteria-expressed and nanocarrier-delivered RNA pesticide for *Myzus persicae* control. J. Pest Sci. 96, 181–193. doi: 10.1007/s10340-022-01485-5

[B31] Ma Y.-F. Zhao Y.-Q. Zhou Y.-Y. Feng H.-Y. Gong L.-L. Zhang M.-Q.. (2024). Nanoparticle-delivered RNAi-based pesticide target screening for the rice pest white-backed planthopper and risk assessment for a natural predator. Sci. Total Environ. 926, 171286. doi: 10.1016/j.scitotenv.2024.171286 38428617

[B32] Ma Z. Zheng Y. Chao Z. Chen H. Zhang Y. Yin M.. (2022). Visualization of the process of a nanocarrier-mediated gene delivery: stabilization, endocytosis and endosomal escape of genes for intracellular spreading. J. Nanobiotechnology 20, 124. doi: 10.21203/rs.3.rs-1255599/v1 35264206 PMC8905852

[B33] Martins M. R. Nascimento A. F. S. de Sena Fernandes M. E. Tronto J. da Fonseca L. F. Fernandes F. L. (2024). Chlorantraniliprole mediating the survival and behavior of adults of *Tuta absoluta* (Meyrick)(Lepidoptera: Gelechiidae) in tomato. Int. J. Trop. Insect Sci. 44, 2879–2888. doi: 10.1007/s42690-024-01392-5

[B34] Nozad-Bonab Z. Hejazi M. J. Iranipour S. Arzanlou M. Biondi A. (2021). Lethal and sublethal effects of synthetic and bio-insecticides on *Trichogramma brassicae* parasitizing Tuta absoluta. PloS One 16, e0243334. 34329292 10.1371/journal.pone.0243334 PMC8323930

[B35] Pang R. Xing K. Yuan L. Liang Z. Chen M. Yue X.. (2021). Peroxiredoxin alleviates the fitness costs of imidacloprid resistance in an insect pest of rice. PloS Biol. 19, e3001190. doi: 10.1371/journal.pbio.3001190 33844686 PMC8062100

[B36] Roditakis E. Steinbach D. Moritz G. Vasakis E. Stavrakaki M. Ilias A.. (2017). Ryanodine receptor point mutations confer diamide insecticide resistance in tomato leafminer, *Tuta absoluta* (Lepidoptera: Gelechiidae). Insect Biochem. Mol. Biol. 80, 11–20. doi: 10.1016/j.ibmb.2016.11.003 27845250

[B37] Roditakis E. Vasakis E. García-Vidal L. del-Rosario-Martínez-Aguirre M. Rison J. L. Haxaire-Lutun M. O.. (2018). A four-year survey on insecticide resistance and likelihood of chemical control failure for tomato leaf miner *Tuta absoluta* in the European/Asian region. J. Pest Sci. 91, 421–435. doi: 10.1007/s10340-017-0900-x

[B38] Silva T. Silva W. Campos M. Silva J. Ribeiro L. Siqueira H. (2016). Susceptibility levels of *Tuta absoluta* (Meyrick)(Lepidoptera: Gelechiidae) to minor classes of insecticides in Brazil. Crop Prot. 79, 80–86. doi: 10.1016/j.cropro.2015.10.012

[B39] Stavrakaki M. Ilias A. Ioannidis P. Vontas J. Roditakis E. (2022). Investigating mechanisms associated with emamectin benzoate resistance in the tomato borer Tuta absoluta. J. Pest Sci.. 95, 1163–1177. doi: 10.1007/s10340-021-01448-2

[B40] Sun Y. Liu S. T. Ling Y. Wang L. Ni H. Guo D.. (2023). Insecticide resistance monitoring of *Cnaphalocrocis medinalis* (Lepidoptera: Pyralidae) and its mechanism to chlorantraniliprole. Pest Manage. Sci. 79, 3290–3299. doi: 10.1002/ps.v79.9 37127919

[B41] Sun H. Wang S. Liu C. Hu W. K. Liu J. W. Zheng L. J.. (2024). Risk assessment, fitness cost, cross-resistance, and mechanism of tetraniliprole resistance in the rice stem borer, Chilo suppressalis. Insect Sci. 31, 835–846. doi: 10.1111/1744-7917.13282 37846895

[B42] Tchouakui M. Riveron Miranda J. Mugenzi L. M. Djonabaye D. Wondji M. J. Tchoupo M.. (2020). Cytochrome P450 metabolic resistance (*CYP6P9a*) to pyrethroids imposes a fitness cost in the major African malaria vector Anopheles funestus. Heredity 124, 621–632. doi: 10.1038/s41437-020-0304-1 32157181 PMC7171194

[B43] Ullah F. Gul H. Tariq K. Hafeez M. Desneux N. Song D. (2023). Silencing of Cytochrome P450 genes *CYP6CY14* and *CYP6DC1* in *Aphis gossypii* by RNA interference enhances susceptibility to clothianidin. Entomologia Generalis 43, 669–678. doi: 10.1127/entomologia/2023/2002

[B44] Ullah F. Güncan A. Gul H. Hafeez M. Zhou S. Wang Y.. (2024). Spinosad-induced intergenerational sublethal effects on *Tuta absoluta*: biological traits and related genes expressions. Entomologia Generalis 44, 395–404. doi: 10.1127/entomologia/2024/2452

[B45] Ullah F. Murtaza G. Li X. Gul H. Wang Y. Zhao S.. (2025). Selection-induced spinosad resistance and associated fitness costs in *tuta absoluta*: A key invasive tomato pest. Agronomy 15, 358. doi: 10.3390/agronomy15020358

[B46] Wang X. Ji S. Bi S. Tang Y. Zhang G. Yan S.. (2023). A promising approach to an environmentally friendly pest management solution: nanocarrier-delivered dsRNA towards controlling the destructive invasive pest Tuta absoluta. Environ. Science: Nano 10, 1003–1015.

[B47] Xu C. Zhang Z. Cui K. Zhao Y. Han J. Liu F.. (2016). Effects of sublethal concentrations of cyantraniliprole on the development, fecundity and nutritional physiology of the black cutworm *Agrotis ipsilon* (Lepidoptera: Noctuidae). PloS One 11, e0156555. doi: 10.1371/journal.pone.0156555 27249654 PMC4889041

[B48] Yan S. Ren B. Zeng B. Shen J. (2020). Improving RNAi efficiency for pest control in crop species. BioTechniques 68, 283–290. doi: 10.2144/btn-2019-0171 32202134 PMC7252490

[B49] Yan S. Yin M.-Z. Shen J. (2022). Nanoparticle-based nontransformative RNA insecticides for sustainable pest control: mechanisms, current status and challenges. Entomol. Gen. 43, 21–30. doi: 10.1127/entomologia/2022/1618

[B50] Zeng B. Liu Y. T. Zhang W. J. Feng Z. R. Wu S. F. Gao C. F. (2022). Inheritance and fitness cost of buprofezin resistance in a near-isogenic, field-derived strain and insecticide resistance monitoring of *Laodelphax striatellus* in China. Pest Manage. Sci. 78, 1833–1841. doi: 10.1002/ps.v78.5 35048493

[B51] Zhang G. F. Xian X. Q. Zhang Y. B. Liu W. X. Liu H. Feng X. D.. (2021). Outbreak of the South American tomato leafminer, *Tuta absoluta*, in the Chinese mainland: Geographic and potential host range expansion. Pest Manage. Sci. 77, 5475–5488. doi: 10.1002/ps.v77.12 34351686

[B52] Zhang X. Zhang J. Zhu K. (2010). Chitosan/double-stranded RNA nanoparticle-mediated RNA interference to silence chitin synthase genes through larval feeding in the African malaria mosquito (*Anopheles Gambiae*). Insect Mol. Biol. 19, 683–693. doi: 10.1111/j.1365-2583.2010.01029.x 20629775

