# Corrigendum: Nanocarrier-mediated RNAi of *CYP9E2* and *CYB5R* enhance susceptibility of invasive tomato pest, *Tuta absoluta* to cyantraniliprole

**DOI:** 10.3389/fpls.2025.1631832

**Published:** 2025-06-25

**Authors:** Farman Ullah, Guru-Pirasanna-Pandi G, Hina Gul, Rudra Madhab Panda, Ghulam Murtaza, Zhijun Zhang, Jun Huang, Xiaowei Li, Nicolas Desneux, Yaobin Lu

**Affiliations:** ^1^ State Key Laboratory for Quality and Safety of Agro-Products, Key Laboratory of Biotechnology in Plant Protection of MOA of China and Zhejiang Province, Institute of Plant Protection and Microbiology, Zhejiang Academy of Agricultural Sciences, Hangzhou, China; ^2^ Protection Division, ICAR-National Rice Research Institute, Cuttack, Odisha, India; ^3^ MARA Key Laboratory of Surveillance and Management for Plant Quarantine Pests, College of Plant Protection, China Agricultural University, Beijing, China; ^4^ Université Côte d’Azur, INRAE, CNRS, UMR ISA, Nice, France

**Keywords:** resistance evolution, RNA interference, biological traits, lepidoptera, gene expression

In the published article, there was an error in the article title. Instead of “Nanocarrier-mediated RNAi of *CYP9E2* and *CYB5R* enhance susceptibility of invasive tomato pest, *Tuta absoluta* to cyantraniliprole”, it should be “Nanocarrier-mediated RNAi of *CYP9A306* and *CYB5R* enhances susceptibility of invasive tomato pest, *Tuta absoluta* to cyantraniliprole”.

In the published article, there was an error. Throughout the manuscript, the gene name “*CYP9E2, CYP92, and CYP9E22*” are wrong. All these three names should be replaced by the correct name “*CYP9A306”*. Also, the word “ds*CYP9E2*/SPc” should be revised as “ds*CYP9A306*/SPc”.

In the published article, there was an error in **Table 1** as published. In **Table 1**, the gene name “*CYP9E2*” is wrong. It should be replaced by the correct name “*CYP9A306”*. Also, the word “ds*CYP9E2*/SPc” should be revised as “ ds*CYP9A306*/SPc. The corrected **Table 1** and its caption “Table 1. Primer sequences used for RT-qPCR and dsRNA synthesis” appears below.

**Table 1:** Primer sequences used for RT-qPCR and dsRNA synthesis.

Primer name	Forward sequence	Reverse sequence
*CYP9A306*	CGAGGTGAAAATCATGGCGT	CAGTGTCCACCCTTCATCCT
*CYB5R*	CGAGAGCGGGAAAATTGAGG	CGACTGTTCTTGGTGACGTC
*RPL28*	TCAGACGTGCTGAACACACA	GCCAGTCTTGGACAACCATT
*TaEF1α*	GAAGCCTGGTATGGTTGTCGT	GGGTGGGTTGTTCTTTGTG
*dsEGFP*	TAATACGACTCACTATAGGGAAGTTCAGCGTGTCCGGCGAGG	TAATACGACTCACTATAGGGCACCTTGATGCCGTTCTTCTGC
ds*CYP9A306*	taatacgactcactatagggTCCTTCTTCACGAGTTGGCT	taatacgactcactatagggACGTTGAAGGTGGAGGTGTC
ds*CYB5R*	taatacgactcactatagggTCGTGTAGTGAGCAAATCGC	taatacgactcactatagggTGTCGTCTTCTTTCGCAATG

In the published article, there was an error in **Figure 2** as published. In **Figure 2**, the gene name “*CYP9E2*” is wrong. It should be replaced by the correct name “*CYP9A306”*. The corrected **Figure 2** and its caption “Figure 2: Relative expression levels of *CYP9A306* and *CYB5R* genes in cyantraniliprole-resistant (CyanRS) and susceptible (SS) strains of *Tuta absoluta*. Data presented as mean ± SE of the three independent biological replicates. The asterisks **** show significant differences at *P* < 0.0001, based on Student’s t-test.” appear below.

**Figure 2:**
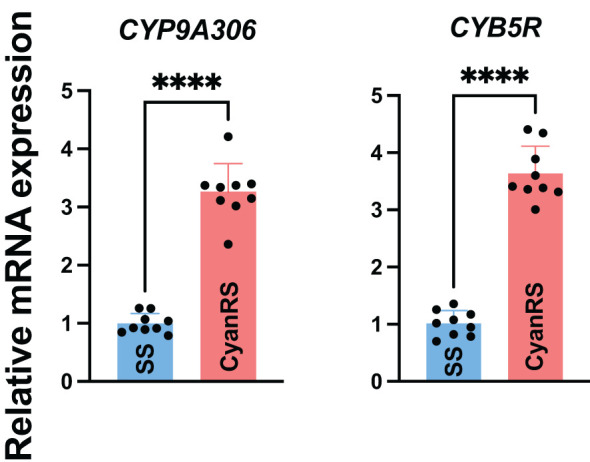
Relative expression levels of *CYP9A306* and *CYB5R* genes in cyantraniliprole-resistant (CyanRS) and susceptible (SS) strains of *Tuta absoluta*. Data presented as mean ± SE of the three independent biological replicates. The asterisks **** show significant differences at *P* < 0.0001, based on Student’s t-test.

In the published article, there was an error in **Figure 3** as published. In **Figure 3**, the gene name “*CYP9E2*” is wrong. It should be replaced by the correct name “*CYP9A306”*. The corrected **Figure 2** and its caption “Figure 3: Phylogenetic and motif analysis of the *CYB5R* (a, b) and *CYP9A306* (c, d) in lepidopteran insect species.” appear below.

**Figure 3:**
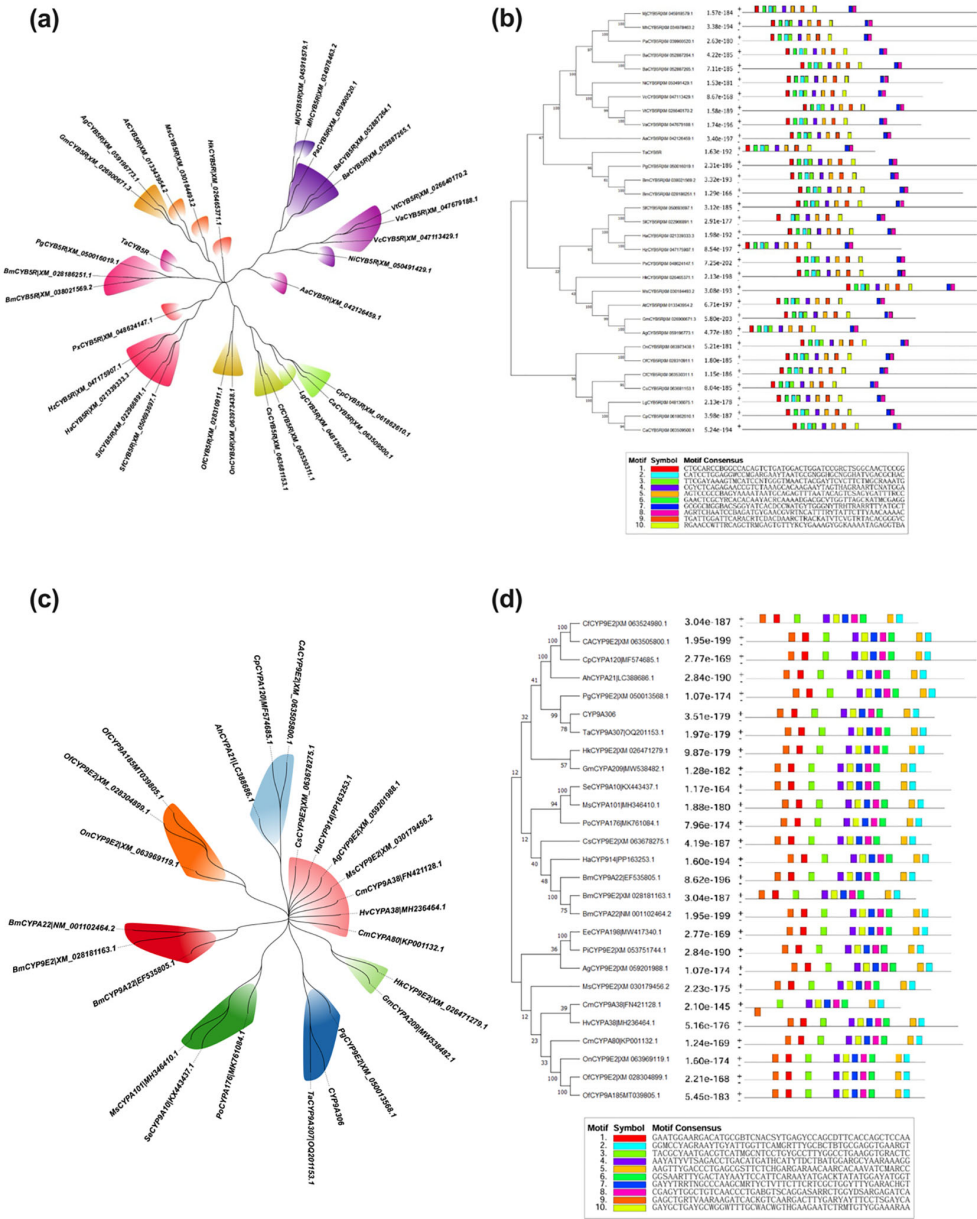
Phylogenetic and motif analysis of the *CYB5R* **(a, b)** and *CYP9A306* **(c, d)** in lepidopteran insect species.

In the published article, there was an error in **Figure 4** as published. In **Figure 4**, the gene name “*CYP9E2*” is wrong. It should be replaced by the correct name “*CYP9A306”*. Also, the word “ds*CYP9E2*/SPc” should be revised as “ds*CYP9A306*/SPc”. The corrected **Figure 4** and its caption “Figure 4: Nanocarrier-mediated RNAi of *CYP9A306* and *CYB5R* genes increase the sensitivity of the cyantraniliprole-resistant strain (CyanRS) of *Tuta absoluta* against cyantraniliprole. (a) Schematic diagram of nanocarrier-mediated RNA inference. (b) Relative mRNA expression level of *CYP9A306* and *CYB5R* genes in CyanRS and SS populations of *Tuta absoluta*. Data presented as mean ± SE of the three independent biological replicates. (c) The activity of cytochrome P450 enzyme among CyanRS and SS populations of *Tuta absoluta*. (d) Mortality rates (%) of cyantraniliprole-resistant strain of *Tuta absoluta* at 48 h of feeding on ds*CYP9A306*/SPc, ds*CYB5R*/SPc, ds*EGFP*/SPc and DEPC water after exposure to the LC_50_ of cyantraniliprole. Different lowercase letters represent significant differences at *P*<0.001 level (one-way analysis of variance (ANOVA) with Tukey’s *post hoc* test.” appear below.

**Figure 4:**
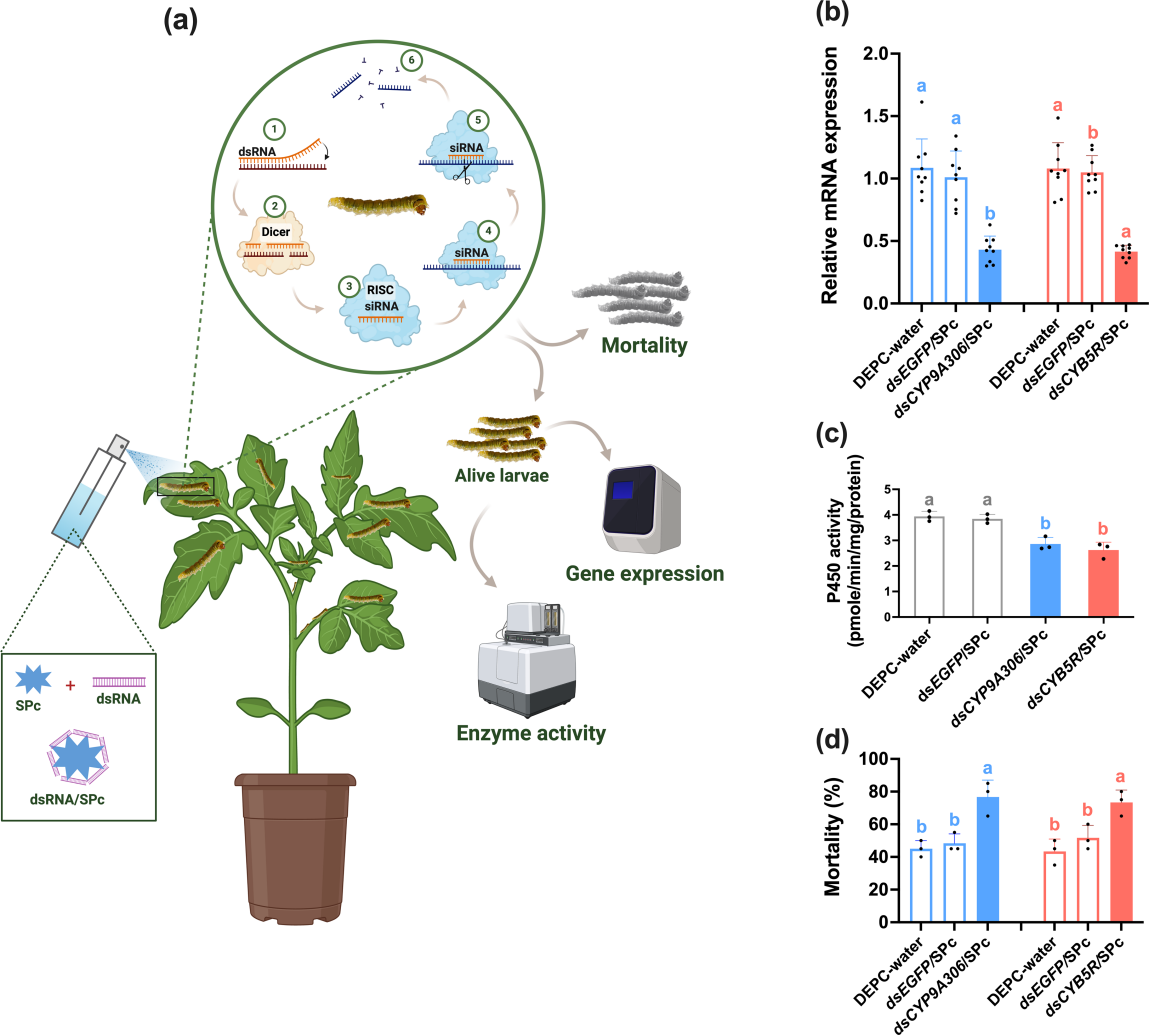
Nanocarrier-mediated RNAi of *CYP9A306* and *CYB5R* genes increase the sensitivity of the cyantraniliprole-resistant strain (CyanRS) of *Tuta absoluta* against cyantraniliprole. **(a)** Schematic diagram of nanocarrier-mediated RNA inference. **(b)** Relative mRNA expression level of *CYP9A306* and *CYB5R* genes in CyanRS and SS populations of *Tuta absoluta*. Data presented as mean ± SE of the three independent biological replicates. **(c)** The activity of cytochrome P450 enzyme among CyanRS and SS populations of *Tuta absoluta*. **(d)** Mortality rates (%) of cyantraniliprole-resistant strain of *Tuta absoluta* at 48 h of feeding on ds*CYP9A306*/SPc, ds*CYB5R*/SPc, ds*EGFP*/SPc and DEPC water after exposure to the LC_50_ of cyantraniliprole. Different lowercase letters represent significant differences at *P*<0.001 level (one-way analysis of variance (ANOVA) with Tukey’s post hoc test.

In the published article, there was an error in **Supplementary Table 1**. The gene name “*CYP9E2*” is wrong. It should be replaced by the correct name “*CYP9A306*”.

The authors apologize for these errors and state that this does not change the scientific conclusions of the article in any way. The original article has been updated.

