# Supplementary material for: Nanocarrier-mediated RNAi of CYP9A306 and CYB5R enhances susceptibility of invasive tomato pest, Tuta absoluta to cyantraniliprole
Source: Front Plant Sci. 2025 Apr 28;16:1573634. doi: 10.3389/fpls.2025.1573634 (PMC12066504; doi:10.3389/fpls.2025.1573634)
Supplement: Supplementary file 1 [file Table1.docx]

**Table S1:** Phylogenetic analysis of *CYB5R* and *CYP9A306* in different insect species.

| **Genes** | **Species Name** |
| --- | --- |
| **Phylogenetic analysis of *CYB5R* gene** | |
| *PgCYB5R\|XM_050016019.1* | *Pectinophora gossypiella* |
| *VtCYB5R\|XM_026640170.2* | *Vanessa tameamea* |
| *PxCYB5R\|XM_048624147.1* | *Plutella xylostella* |
| *MjCYB5R\|XM_045918579.1* | *Maniola jurtina* |
| *MsCYB5R\|XM_030184493.2* | *Manduca sexta* |
| *OnCYB5R\|XM_063973438.1* | *Ostrinia nubilalis* |
| *OfCYB5R\|XM_028310911.1* | *Ostrinia furnacalis* |
| *CfCYB5R\|XM_063530311.1* | *Cydia fagiglandana* |
| *CsCYB5R\|XM_063681153.1* | *Cydia strobilella* |
| *CpCYB5R\|XM_061862610.1* | *Cydia pomonella* |
| *LgCYB5R\|XM_048136075.1* | *Leguminivora glycinivorella* |
| *CaCYB5R\|XM_063509500.1* | *Cydia amplana* |
| *HkCYB5R\|XM_026465371.1* | *Hyposmocoma kahamanoa* |
| *MhCYB5R\|XM_034978463.2* | *Maniola hyperantus* |
| *BmCYB5R\|XM_038021569.2* | *Bombyx mori* |
| *BmCYB5R\|XM_028186251.1* | *Bombyx mandarina* |
| *AtCYB5R\|XM_013343954.2* | *Amyelois transitella* |
| *GmCYB5R\|XM_026900671.3* | *Galleria mellonella* |
| *SfCYB5R\|XM_050693697.1* | *Spodoptera frugiperda* |
| *BaCYB5R\|XM_052887264.1* | *Bicyclus anynana* |
| *HaCYB5R\|XM_021339333.3* | *Helicoverpa armigera* |
| *BaCYB5R\|XM_052887265.1* | *Bicyclus anynana* |
| *AgCYB5R\|XM_059196773.1* | *Achroia grisella* |
| *HzCYB5R\|XM_047175907.1* | *Helicoverpa zea* |
| *SlCYB5R\|XM_022966891.1* | *Spodoptera litura* |
| *PaCYB5R\|XM_039900520.1* | *Pararge aegeria* |
| *AaCYB5R\|XM_042126459.1* | *Aricia agestis* |
| *NoCYB5R\|XM_050491429.1* | *Nymphalis io* |
| *VaCYB5R\|XM_047679188.1* | *Vanessa atalanta* |
| *VcCYB5R\|XM_047113429.1* | *Vanessa cardui* |
| **Phylogenetic analysis of *CYP9A306* gene** | |
| *CYP9A306* | *Tuta absoluta* |
| *TaCYP9A307\|OQ201153.1* | *Tuta absoluta* |
| *BmCYP9E2\|XM_028181163.1* | *Bombyx mandarina* |
| *BmCYP9A22\|EF535805.1* | *Bombyx mori* |
| *HkCYP9E2\|XM_026471279.1* | *Hyposmocoma kahamanoa* |
| *BmCYPA22\|NM_001102464.2* | *Bombyx mori* |
| *CfCYP9E2\|XM_063524980.1* | *Cydia fagiglandana* |
| *EeCYPA198\|MW417340.1* | *Ephestia elutella* |
| *OfCYP9E2\|XM_028304899.1* | *Ostrinia furnacalis* |
| *OfCYP9A185MT039805.1* | *Ostrinia furnacalis* |
| *AhCYPA21\|LC388686.1* | *Adoxophyes honmai* |
| *OnCYP9E2\|XM_063969119.1* | *Ostrinia nubilalis* |
| *MsCYP9E2\|XM_030179456.2* | *Manduca sexta* |
| *CmCYP9A38\|FN421128.1* | *Cnaphalocrocis medinalis* |
| *PgCYP9E2\|XM_050013568.1* | *Pectinophora gossypiella* |
| *HaCYP914\|PP163253.1* | *Helicoverpa armigera* |
| *CaCYP9E2\|XM_063505800.1* | *Cydia amplana* |
| *HvCYPA38\|MH236464.1* | *Heortia vitessoides* |
| *PoCYPA176\|MK761084.1* | *Plecoptera oculata* |
| *AgCYP9E2\|XM_059201988.1* | *Achroia grisella* |
| *PiCYP9E2\|XM_053751744.1* | *Plodia interpunctella* |
| *SeCYP9A10\|KX443437.1* | *Spodoptera exigua* |
| *CmCYPA80\|KP001132.1* | *Cnaphalocrocis medinalis* |
| *MsCYPA101\|MH346410.1* | *Mythimna separata* |
| *CpCYPA120\|MF574685.1* | *Cydia pomonella* |
| *CsCYP9E2\|XM_063678275.1* | *Cydia strobilella* |
| *GmCYPA209\|MW538482.1* | *Grapholita molesta* |
